# Microdosing psilocybin for major depressive disorder: study protocol for a phase II double-blind placebo-controlled randomised partial crossover trial

**DOI:** 10.1192/bjo.2025.10968

**Published:** 2026-02-16

**Authors:** Zeina Beidas, Anya Ragnhildstveit, Adam Blackman, Thomas Anderson, Emily Fewster, Omer A. Syed, Valentyne Sobolenko, Ismail Kaan Kanca, Magdalena Jaglinska, Tatiana Son, Norman Farb, Rotem Petranker

**Affiliations:** Canadian Centre for Psychedelic Science, Toronto, Ontario, Canada; Center for Psychedelic Research, Pneuma Science, Draper, Utah, USA; Department of Psychiatry, University of Cambridge, Cambridge, UK; Department of Psychiatry and Behavioral Sciences, Baylor College of Medicine, Houston, Texas, USA; Psychedelic Studies Research Program, University of Toronto, Mississauga, Ontario, Canada; Department of Psychiatry, University of Toronto, Toronto, Ontario, Canada; Department of Psychology, University of Toronto, Toronto, Ontario, Canada; Institute of Medical Science, University of Toronto, Toronto, Ontario, Canada; Department of Experimental Psychology, University College London, UK; Canadian Cancer Trials Group, Queen’s University, Kingston, Ontario, Canada; Department of Surgery, McMaster University, Hamilton, Ontario, Canada; Remedy Institute, Toronto, Ontario, Canada; Centre for Addiction and Mental Health, Toronto, Ontario, Canada; Department of Psychological & Brain Sciences, University of Toronto, Mississauga, Ontario, Canada; Graduate Department of Psychological Clinical Science, University of Toronto, Scarborough, Ontario, Canada; MettaScience Inc., Toronto, Ontario, Canada

**Keywords:** Psilocybin, microdosing, major depressive disorder, randomised controlled trial

## Abstract

**Background:**

Major depressive disorder (MDD) is the leading cause of disability worldwide, affecting roughly 322 million people. Recently, doses of psilocybin have shown promise in treating mood disorders, sparking interest in other dosing practices. According to anecdotal reports and observational studies, microdosing psilocybin yields benefits to mental health; however, rigorously controlled trials have failed to produce compelling evidence for this.

**Aims:**

To conduct a phase II, double-blind, placebo-controlled, randomised partial crossover trial to compare microdosing psilocybin to placebo for MDD, evaluating its safety, tolerability and preliminary antidepressant effects.

**Method:**

Forty adults with MDD will be randomised to four doses of psilocybin (2 mg) or placebo (maltodextrin) once weekly over 4 weeks, then four doses of psilocybin (2 mg) once weekly for an additional 4 weeks. The primary efficacy end-point will be change in depression symptoms, as measured at baseline (0 weeks), after the experimental phase (4 weeks), and after the open-label phase (8 weeks). A battery of mood, well-being, attention, creativity, mindfulness and pro-sociality measures will be administered at each time point. Follow-ups will occur every 6 months for up to 2 years after the trial start date, as part of a long-term extension study.

**Results:**

The results of the primary outcome of this trial will be published as a manuscript in a peer-reviewed science or medical journal regardless of the magnitude or direction of effect.

**Conclusions:**

Findings will inform future research on microdosing psilocybin for MDD, regarding dose regimens, effect sizes and expectancy bias. Findings will also facilitate discussions on the comparable benefits of sub- versus threshold doses of psilocybin and the therapeutic value of radically altered perception.

**Trial registration:**

ClinicalTrials.gov identifier: NCT05259943.

Major depressive disorder (MDD) is the most common psychiatric illness worldwide, and the leading cause of disability, affecting roughly 322 million people or 4.4% of the global population.^1^ While some people significantly benefit from pharmacological treatment for MDD, more than a third fail to respond fully at adequate dose and duration.^2^ Of those who do respond, up to 60% report emotional blunting and other undesired reactions that may lead to medication non-adherence and poor quality of life.^3,4^ Novel interventions that mitigate such adverse effects and promote mental health are therefore urgently needed.

In recent years, there has been renewed interest in classic psychedelics, like psilocybin (‘magic mushrooms’) and LSD (‘acid’), due to their transdiagnostic potential to treat a variety of psychiatric disorders, including MDD.^5^ Psilocybin, in particular, has been granted a ‘Breakthrough Therapy’ designation for treatment-resistant depression by the Food and Drug Administration.^6^ Dosing plays a role in determining the hallucinogenic effects, and ranges from ‘microdoses’ to ‘macrodoses’. Microdosing refers to the use of psychedelics at low doses, commonly 1/10th of a therapeutic dose, which does not occasion perceptual or psychoactive effects, like mystical experiences,^7,8^ nor impairs normal cognitive functioning.^9^ Microdoses are self-administered over an extended period of time, with the most common regimen for psilocybin being 0.1–0.3 g dried mushroom, taken 1–3 times per week, for 1 week to several months.^8–15^

Microdosing is an appealing therapeutic model, as it circumvents the need to induce non-ordinary states of consciousness that might otherwise be intense and challenging to navigate.^16,17^ This is a particular consideration for individuals with emotion dysregulation, a core psychopathological feature of MDD, who may lack the necessary self-regulation skills to handle difficult and/or distressing psychedelic content (e.g. trauma or life issues) and states (e.g. confusion, paranoia or troubling visions), even when situated in a medically supervised and supportive setting.^17,18^ Research suggests that microdosing may help to reduce depressive symptoms by enhancing emotional resilience, increasing self-efficacy and promoting cognitive flexibility, which can disrupt the rigid thought patterns often associated with MDD.^19,20^ Other studies highlight potential benefits such as improved problem-solving abilities and enhanced neural plasticity, which could contribute to long-term improvements in mood regulation.^21^ However, the few prospective studies that have examined the potential benefits of microdosing for mood have not formally assessed MDD and have been largely observational to date (i.e. online surveys or open-label field studies).^7,10,12,22–28^ Unfortunately, these studies lack robust controls, primarily use convenience sampling and are likely tainted by expectancy effects. This makes them vulnerable to confirmation bias and minimises the validity of results.^10,29^

Furthermore, the difficulty of blinding participants has constrained the conduct of randomised controlled trials (RCTs), given the inherent mind-altering properties of high-dose psychedelics^30^. However, even though blinding should be less of a challenge in microdosing versus large dose studies due to the subtle nature of its effects, psychological expectancy may still play a role. Some studies found similar reported benefits between microdosing and placebo groups despite blinding.^31,32^ These studies, however, are limited by small sample sizes and methodological issues in assessing blinding,^32^ meaning that our confidence in their findings remains limited. Overall, despite the potential of microdosing for enhancing mood, cognition and well-being and other areas such as perception and creativity, findings on the whole are mixed.^33–35^ Thus, there is a need for well-controlled studies to effectively converge on the efficacy of microdosing psychedelics for MDD and beyond. We designed a phase II, double-blind, placebo-controlled, randomised partial crossover trial to compare microdosing psilocybin to placebo for MDD, with the aim of evaluating its safety, tolerability and antidepressant effects. We will also explore other domains related to well-being, attention, creativity, mindfulness and pro-sociality. To our knowledge, this trial will be one of the first and largest prospective trials to date to evaluate microdosing psilocybin for MDD.

## Objectives

### Primary objective

The primary objective of this study is to test the effect of low-dose psilocybin on depressive symptoms at baseline, after 4 weeks of either psilocybin or placebo, and after 8 weeks of either 4 or 8 weeks of psilocybin and longitudinally for up to 2 years.

### Secondary objective

The secondary objective is to study the effect of microdosing on attention, creativity, pro-sociality, trait, state and cognitive measures of mood, anxiety, well-being (general self-efficacy, dysfunctional attitudes, sleep, quality of life, pain inventory and personality), mindfulness, mystical experiences, interoceptive awareness and qualitative experiences. See Supplementary material available at https://doi.org/10.1192/bjo.2025.10968.

### Safety objective

We aim to test the safety and tolerability of microdosing by measuring effects on suicidal ideation and behaviour, vital signs, sobriety and depressive symptom severity.

## Method

### Study design

**Fig. 1:**
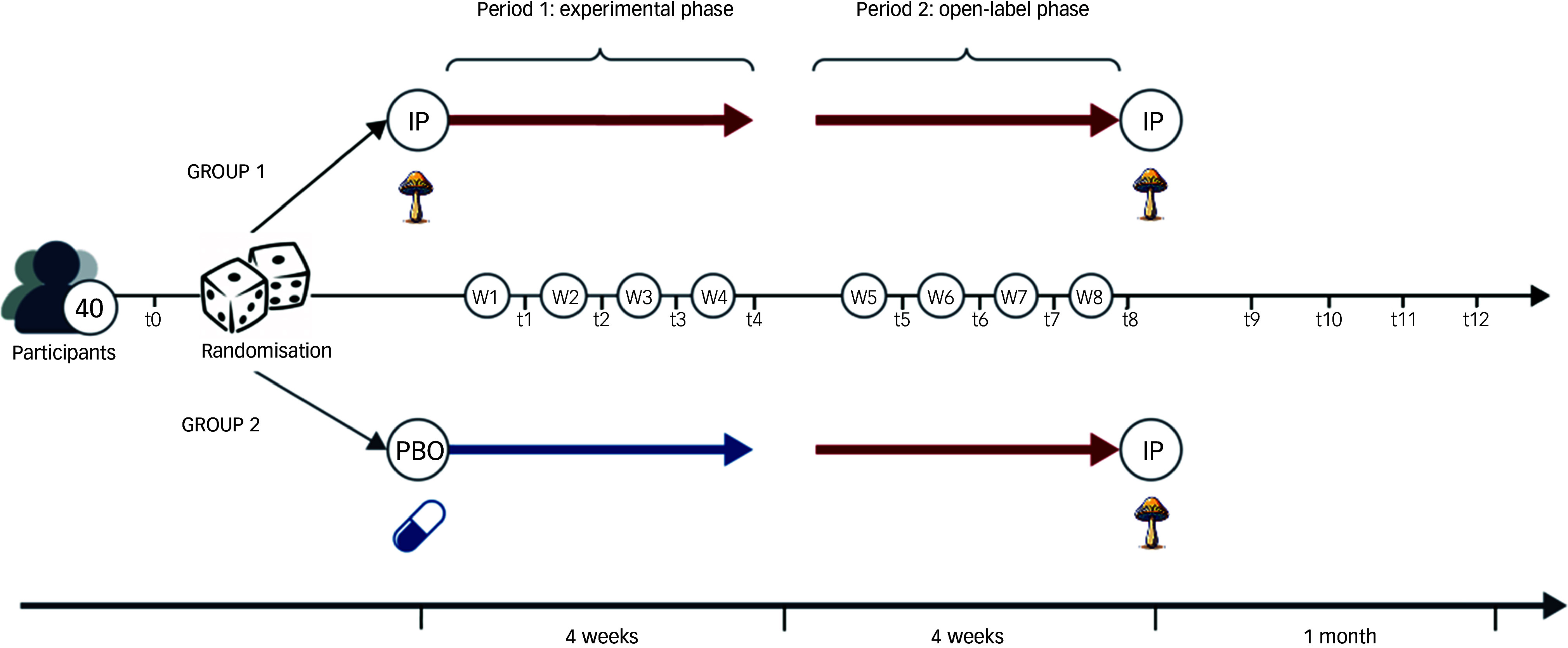
Visual depiction of the 2 × 1 crossover design over 8 weeks. IP, psilocybin mushroom; PBO, inactive placebo.

This is a single-site, phase II, double-blind, triple-masked, inactive-placebo-controlled, randomised partial crossover trial. It consists of an experimental phase (4 weeks) that switches to an open-label phase (4 weeks), with no medication washout in between. This defines the treatment period (8 weeks). Forty participants with clinically diagnosed MDD will be randomised to one of two groups in a 1:1 allocation ratio: 2 mg psilocybin once weekly over 4 weeks, followed by 2 mg psilocybin once weekly for an additional 4 weeks (treatment group: IP_phase 1_ → IP_phase 2_) OR 2 mg inactive placebo (maltodextrin) once weekly over 4 weeks, followed by 2 mg psilocybin mushroom once weekly for an additional 4 weeks (control group: PBO_phase 1_ → IP_phase 2_). See Fig. 1 for a visual description of interventions for each group. We favoured a crossover design to reduce intra-subject variability from the comparison between groups, to minimise the risk of potential confounds (with each participant serving as their own control),^36^ and to increase statistical power and efficiency (by reducing the number of participants required to detect a significant effect).^37,38^ The partial 2 × 1 crossover design was chosen to better model the response to psilocybin mushroom, including the anticipated carryover effect from the experimental to open-label phase, relative to 2 × 2 crossover designs.^39^ It also allows us to test the role of the placebo, if any, on therapeutic outcome in the psilocybin group once they move into open-label, and in the placebo group once they begin taking psilocybin.

### Study procedures

#### Participant recruitment

Participants will be recruited via local signage (e.g. coffee shops), mailing lists (e.g. psychedelic newsletters) and social media adverts (e.g. Facebook, LinkedIn) in Ontario, Canada. Recruiting material will direct prospective participants to complete an online pre-screening questionnaire which contains a brief description of the study and an exhaustive list of questions on demographics, medical and psychiatric history, treatment history and substance use. Those who pass the online questionnaire will be contacted by the study team to undergo a pre-screening call. If a prospective participant satisfies the inclusion and exclusion criteria of pre-screening, they will be asked to provide proof of a recent medical diagnosis for MDD or be invited to be evaluated for a diagnosis by the study psychiatrist. Upon confirmation of diagnosis, participants will be sent an email containing the informed consent form and invited to a screening visit. Those who provide written informed consent at the start of the screening visit and meet full eligibility criteria will be invited to participate in the study.

#### Eligibility criteria

Inclusion criteria will include adults (aged 18–65) with mild to moderate MDD, as diagnosed in-person by an independent physician and confirmed by Structured Clinical Interview for DSM-5 (SCID-5) scores.^40^ The severity of depression will be determined by a study psychiatrist, who will assess the number of symptoms present and the degree to which they caused distress in participants. We chose mild to moderate depression as a relatively lower risk population compared with individuals with severe depression, given the unknown safety risks of psychedelic use. A history of mania or hypomania as assessed by the SCID and psychiatric interview will be ruled out, provided their status as known contraindications to classic psychedelic use.^41^ Further, participants with a past suicide attempt and/or current active suicidal ideation (i.e. currently endorse suicidal thoughts with urges to act on them) as assessed by the SCID, psychiatric interview and Columbia-Suicide Severity Rating Scale (C-SSRS)^42^ will be excluded given the paucity of evidence on microdosing psilocybin and suicidality and our aim to include individuals with mild to moderate MDD.^43^ Individuals taking psychotropics, opioids, serotonin medications or receiving any form of psychotherapy will be excluded. Participants must also refrain from making any major changes in lifestyle activities (e.g. no major changes in coffee use, no new therapies and no night shifts) and must agree to not consume cannabis or alcohol within 24 h of each dosing session. These exclusions are implemented to reduce potential confounds to the study results. A complete list of inclusion and exclusion criteria is provided in Table 1.

**Table 1:** Full list of inclusion/exclusion criteria

Inclusion criteria	
Consent	-Have given written informed consent. -Have a high school level of education. -Be fluent in speaking and reading English. -If of childbearing potential, must have a negative pregnancy test at study entry and use adequate birth control through 10 days after the last experimental session. -Agree to refrain from taking non-prescription medication, nutritional supplements or herbal supplements for one week before each psilocybin session, except as approved by the research team. Exceptions will be evaluated by the research team and will include acetaminophen, non-steroidal anti-inflammatory drugs, and common doses of vitamins and minerals. -Are willing to comply with medication requirements per the protocol. -Have not participated in another investigational study within 60 days prior to the screening visit.
Demographics	-Be 18 to 65 years old. -Any gender.
Clinical characteristics	-Have a pre-existing diagnosis of mild or moderate MDD or receive this diagnosis before screening.
Lifestyle requirements	-Agree to consume the same amount of caffeine-containing beverage on psilocybin session days, not exceeding 600 mg/day. -If not a regular caffeine consumer, agree to abstain from caffeine on psilocybin session days. -Agree not to take PRN medications on psilocybin session mornings. -Agree to refrain from starting any new medications. -Agree to refrain from using psychoactive drugs, including alcohol, within 24 h of psilocybin administration. -Refrain from starting any new complementary or alternative medicine practices (e.g. nutrition/diet modifications, supplements, meditation practice, psychotherapy etc). -Agree to refrain from working night shifts.
Exclusion criteria	
Physical health	-Cardiovascular conditions including coronary artery disease, uncontrolled hypertension, angina, atrial fibrillation, TIA (last 6 months), stroke, peripheral/pulmonary vascular disease and other clinically significant structural heart diseases (e.g. cardiac fibrosis, hypertrophic cardiomyopathy). -Blood pressure exceeding 150 systolic and 100 diastolic at screening. -Epilepsy with a history of seizures. -History of cerebral ischaemia, transient ischaemic attack, intracranial aneurysm or arteriovenous malformation. -Clinically significant history of head injury or trauma. -History of cancer. -Unstable medical conditions, severe renal disease (creatinine clearance <40 ml/min), hepatic disease or serious CNS pathology. -Insulin-dependent diabetes or history of hypoglycaemia with oral hypoglycaemic agents. -Pregnant, nursing or is of childbearing potential and not practising effective means of birth control.
Drug use	-Regular use of psychotropic medications including antidepressants, MAO inhibitors, opioids or medications affecting serotonin neurons. -Use of steroids within the past 2 weeks. -Current use of excluded compounds: ergot alkaloids, pimozide, midazolam, triazolam, lovastatin, simvastatin, fentanyl, SAM-e, 5-HTP, L-tryptophan and St. John’s Wort. -Use of psychedelics in the last 6 months. -Had a previous negative experience (i.e. requiring medical attention) with any psychedelic substance. -Have sensitivity to maltodextrin.
Mental health diagnoses	-Current or past DSM-5 diagnosis of schizophrenia, psychotic disorder or bipolar I/II disorder. -First- or second-degree relative with schizophrenia, psychotic disorder (unless substance-induced or due to a medical condition) or bipolar I/II disorder. -Current DSM-5 diagnosis of dissociative disorder, anorexia nervosa, bulimia nervosa, or other psychiatric conditions judged incompatible with safe psilocybin exposure. -Current or past moderate/severe alcohol or drug use disorder (last 5 years, excluding caffeine and nicotine).
Mental health risk	-Current pervasive suicidal ideation determined by Columbia-Suicide Severity Rating Scale. -Past suicide attempt.

MDD, major depressive disorder; PRN, pro re nata; TIA, transient ischemic attack, CNS, central nervous system; MAO, monoamine oxidase; SaM-e, S-adenosylmethionine; 5-HTP, 5-hydroxytryptophan.

#### Drug preparation and administration

The 2 mg psilocybin capsules (PEX010) containing naturally extracted psilocybin mushrooms and 2 mg placebo (maltodextrin) will be provided by Filament Health (Burnaby, British Columbia, Canada), and will be packaged in identical capsules according to good manufacturing practice guidelines. Both capsules will be taken orally. The 2 mg dose is roughly 10% of threshold psilocybin doses that are commonly administered in clinical trials^44,45^ and satisfies the definition of a ‘microdose’ (i.e. not greater than 0.1 mg/kg).^46^ This amount has also been endorsed by psychedelic users who microdose in real-world settings and therefore satisfies ecological validity.^7,8^ The 2 mg fixed-dose psilocybin will not be modified in response to patient preference or symptom trajectory.

#### Participant timeline

Participants enrolled during the screening visit, will be invited to complete 8 weekly experimental sessions on-site, and a weekly online follow-up period for 1 month after. Participants will also be invited to an optional long-term follow-up where they will receive short surveys to complete via email every 6 months for 2 years after the last experimental session. The breakdown of the visits is briefly described in order below:

- **Screening and baseline (V1)**: Participants who meet the pre-screening eligibility criteria, including diagnosis of mild to moderate depression using the SCID, will be invited to screening. Informed consent will be acquired by research staff, and will be followed by physical exams (vitals and neurological tests) and blood tests for drug use and pregnancy (if applicable). Medical history and details regarding concomitant medications (over-the-counter, herbs, supplements and vitamin prescriptions) and reasons for their use will be collected. Participants will also be informed of limitations to lifestyle changes during the trial. If participants pass preliminary assessments (e.g. drug use, medical history and physical exams), they will then complete baseline assessments, including primary measures of depression, well-being, mood, quality of life, emotion, sleep, pain, attention and creativity. Upon passing laboratory assessments, participants will be enrolled. Participants will then be randomised to either the placebo-first or psilocybin group.
- **Experimental sessions and associated post-experimental day surveys (V2–V9)**: Participants will receive either placebo or psilocybin for the first 4 weeks and psilocybin for the last 4 weeks. After capsule administration, participants will complete assessments of depressive symptoms, mood, well-being, pro-sociality and qualitative experiences. Participants will also do attention and creativity tasks only on weeks 1, 4, and 8. Sobriety tests will be conducted at pharmacodynamic peak and prior to release. Participants will be reminded to complete brief surveys assessing mood that will be sent out 3 days after each experimental visit. Visits will be exactly 7 days apart in accordance with Research Ethics Board (REB) approval. Sessions that participants do not attend for any given reason will be counted as missed.
- **Follow-up period (V10)**: After the last experimental session, participants will receive a survey link to complete measures of depressive symptoms and severity once a week for 4 weeks. If a depressive questionnaire has a high score indicating severe depressive symptoms (score of 12 or more on the Quick Inventory of Depressive Symptoms (QIDS) questionnaire)^47^, the study psychiatrist will conduct a full diagnostic interview for depression via phone and refer participants to urgent care if needed.
- **Optional long-term follow-up (LTFU; V11–V14)**: Participants who opt for the optional LTFU will sign a LTFU informed consent form. Participants will receive a survey assessing lifestyle modifications, depression and anxiety symptoms and qualitative experiences every 6 months for 2 years.

See Table 2 for the trial’s schedule of events and Supplementary material B for a day-to-day description of assessments done and their order.

**Table 2:** Trial flow

	Screening	Treatment period 1				Treatment period 2				Follow-up	Extension			
Visit	V1	V2	V3	V4	V5	V6	V7	V8	V9	V10	V11	V12	V13	V14
Visit window (days)	±7 days	7 days	7 days	7 days	7 days	7 days	7 days	7 days	7 days	7–14–21–28 days	6 months	6 months	6 months	6 months
Informed consent and demographics	X													
Childbearing potential	X													
Inclusion and exclusion criteria	X	X	X	X	X	X	X	X	X	X				
Lifestyle modifications	X	X	X	X	X	X	X	X	X	X				
Medical history	X													
Conmedications	X	X	X	X	X	X	X	X	X	X	X	X	X	X
Coffee and tobacco use	X	X	X	X	X	X	X	X	X	X				
Randomisation (enrolment confirmation)	X													
Vitals, body temperature, blood pressure	X	X	X	X	X	X	X	X	X					
Body weight, height	X													
Lab assessments and urinalysis	X													
Physical examination	X													
Urine drug test	X	X				X								
Pregnancy test	X	X				X								
Adverse event/SAE	X	X	X	X	X	X	X	X	X	X				
Drug administration		X	X	X	X	X	X	X	X					
SCID	X				X				X					
QIDS^a^	X	X	X	X	X	X	X	X	X					
C-SSRS	X	X	X	X	X	X	X	X	X					
PHQ-SADS	X	X	X	X	X	X	X	X	X	X	X	X	X	X
UUT, 5-DOT, RAT, insight problems	X	X				X								
GSE		X	X	X	X	X	X	X	X					
DAS-A-17^a^	X	X			X				X					
BFI-II	X				X				X					
FFMQ	X				X				X					
MAIA-2	X				X				X					
MEQ30		X	X	X	X	X	X	X	X					
MRT, SART		X			X				X					
MIE		X			X				X					
IOS		X	X	X	X	X	X	X	X					
SQS	X	X	X	X	X	X	X	X	X					
PANAS^a^	X	X	X	X	X	X	X	X	X					
BPI-SF	X	X	X	X	X	X	X	X	X					
QOLI	X	X	X	X	X	X	X	X	X	X X				
QRSB^a^, QRSPD, QR-SR	X	X	X	X	X	X	X	X	X		X	X	X X	

SAE, severe adverse event; SCID, Structured Clinical Interview for DSM-5; QIDS, Quick Inventory of Depressive Symptomatology; C-SSRS, Columbia-Suicide Severity Rating Scale; PHQ-SADS, Patient Health Questionnaire-Somatic, Anxiety and Depressive Symptoms; UUT, Unusual Uses Task; 5-DOT, Five Dot Problem; RAT, Remote Associates Task; GSE, General Self Efficacy Scale; DAS-A-17, Dysfunctional Attitudes Scales-17 item; BFI-II, Big Five Inventory II; FFMQ, Five Facet Mindfulness Questionnaire; MAIA-2, Multidimensional Assessment of Interoceptive Awareness-2; MEQ-30, Mystical Experience Questionnaire-30 item; MRT, Metronome Response Task; SART, Sustained Attention Reaction Task; MIE, Mind in the Eyes; IOS, Inclusion of Others in the Self scale; SQS, Single Item Sleep Quality Scale; PANAS, Positive and Negative Affective Scale; BPI-SF, Brief Pain Inventory-Short Form; QOLI, Quality of Life Inventory; QRSB, Qualitative Report - social perceptions and sharing; QRSPD, Qualitative Report - self-assessed symptoms and psychological discomfort; QR-SR, Qualitative Report - individual experience/subject responses.

a. Will be sent 3 days after experimental session.

#### Withdrawal criteria

Participants may withdraw from the study freely at any time for any reason. Where known, the reason for withdrawal will be recorded by the investigator. Participants may be withdrawn by the investigator if their safety is compromised, they become uncooperative or no longer meet inclusion criteria. Any withdrawal or adverse event that leads to withdrawal (if applicable) as determined by the investigator will be explained to the participant and documented. If an adverse event was the reason for withdrawal, the investigator will arrange follow-up appointments until the event has resolved or stabilised.

### Outcomes

#### Primary outcomes

The primary outcome will be a change in depressive symptoms, which will be tested using two primary outcome measures. The first, the Patient Health Questionnaire- Somatic, Anxiety and Depressive Symptoms Scale (PHQ-SADS), is a standardised multidimensional 32-item self-report subset of the full PHQ designed to detect the co-occurrence of somatic, anxiety and depressive symptoms.^48^ The response on each of the somatic, anxiety and depressive symptoms will be summed to give a total score for each construct. Higher scores indicate higher symptom load on each given construct. The PHQ-SADS will be completed at every visit.

The second primary outcome measure, SCID, is designed as a brief, structured diagnostic interview for major psychiatric disorders in the DSM-5.^49^ Validation and reliability studies have shown that the psychometric indicators of this instrument are within the parameters of the health status instruments.^50^ This study will use the SCID depression modules to assess the severity of depressive symptoms at session 1, 4 and 8.

#### Safety outcomes

We will assess the severity, incidence, and frequency of adverse effects using the Columbia Suicide Severity Rating Scale (C-SSRS)^42^ at every visit. The lifetime version of the C-SSRS will be used as a screening measure at baseline to ensure no participants have endorsed past suicide attempt or have current active suicidal ideation. The following visits will use the ‘since last visit’ C-SSRS version as a safety measure to ensure there are no changes in suicidal ideation and behaviour. An appearance or increase in suicidal ideation or behaviour from ‘since last visit’ will be considered an adverse event. Additional safety measures include vital signs and three pass/fail sobriety tests to assess the intensity of effects of the IP on balance, motor functioning and coordination relative to baseline (see Supplementary material A). Sobriety tests will be completed before a participant leaves for a break and at the end of every experimental session. Participants will not be discharged until they pass the sobriety tests and are approved for discharge by the investigator. Vital signs will also be measured to assess for any adverse effects. Normal blood pressure values will not exceed 150 systolic over 100 diastolic. Vital signs will be measured at every visit to assess significant deviations from baseline. A significant change in blood pressure will be identified as an adverse event.

#### Secondary and other outcomes

Changes in various other constructs, including but not limited to attention, creativity, prosociality and aspects of well-being, will be measured using well-validated tools.

A full description of all measures and their scoring is provided in Supplementary material B.

#### Strategies to improve adherence

After collection of vitals, medication and study adherence information, participants will take the capsules. After capsule administration, participants will remain in the lab for the duration required to complete all tasks of each experimental session. After all tasks are completed, participants will check-in with the principal investigator for an opportunity to address questions or concerns, if any. All concerns will be addressed appropriately and reasonable changes will be implemented to enhance administration flow and thereby participant adherence. The investigator will also remind participants to complete follow-up surveys. Random psychoactive drug testing will also occur throughout the trial to ensure participants are adhering to protocol requirements.

### Relevant concomitant care

If, during screening, a participant is found to have recently discontinued any psychotropic drugs within the last 6 weeks, there will be a required washout period (at least five times the particular drug and its metabolites’ half-life, plus one week for stabilisation) before the first experimental session to avoid possible drug-drug interaction. All herbal supplements, vitamins, non-prescription medications and prescription medications will be reviewed and approved by the investigator (see Table 1). The medications listed in the exclusion criteria are prohibited during the study and administration will be considered a protocol violation. If participants are found to have met any exclusion criteria, they will be withdrawn from treatment and continue to follow-up.

### Randomisation, masking and code breaking

After completing relevant screening assessments, eligible participants will be randomly assigned to one of the treatment arms. Randomisation will be done via computer-generated random numbers which are sequentially based. Unblinded pharmacists will conduct randomisation and IP handling and will not interact with participants or be present during drug administration to reduce the risk of breaking blind. Medications will have corresponding active or placebo number labels for pharmacists to use for correct allocation to participants based on their trial arm. The allocation information will be stored in a randomisation table in a separate part of an electronic data capture (EDC) software that is locked and only accessible to pharmacists. After allocation, pharmacists will dispense the 2 mg capsules in envelopes with each envelope containing only the participant ID and treatment session. Participants will self-administer the capsules. In the qualitative questionnaires that participants take during every visit, there will be a question probing participants’ guesses of whether they are in the experimental or placebo groups and will be used in analysis to ensure blinding was successful. In emergencies, the investigator will contact a delegate pharmacist to unblind a participant. An investigator should only un-blind a participant when it is vital for immediate medical care or safety, following International Conference on Harmonization (ICH) and Good Clinical Practice (GCP) guidelines, the Declaration of Helsinki and Standard Operating Procedures (SOPs). Unique participant codes ensure that breaking one code doesn‘t compromise the blinding for other participants.

### Data collection and management

An EDC (REDCap version v14.0.21 for Windows; Vanderbilt University Medical Center, Nashville, Tennessee, USA) will be used to store all baseline and outcome data (see Supplementary material A for a complete list of measures). CRFs about inclusion/exclusion criteria, lifestyle modifications, concomitant medication, blood pressure and adverse events for each participant in each visit will be captured. Most trial measures will be self-reported and completed by participants in survey form. The exception is the creativity tasks, which will be administered through Qualtrics due to its time-limit function, and attention tasks, which will be administered through an external application. Physical responses from two creativity tasks (see 5-DOT and insight tasks in Supplementary material A) and Excel files of attention tasks will be transferred onto the EDC.

All data stored on the EDC servers are locked with access restricted to study personnel. Data on the EDC will be attached to a random participant ID and will not contain any personal health identifying information (e.g. name, date of birth). All personal health identifying information will be stored in an encrypted and password-protected Google workspace separate from the study data.

Data from the EDC will be downloaded to password-protected, encrypted machines securely locked at the study trial location. This data will also be stored on the above-mentioned drive. Identifying information will be permanently destroyed at the end of data collection. Data of potential participants who do not meet the inclusion/exclusion criteria will be immediately destroyed.

The investigator will ensure complete data collection for all participants, including those who discontinue treatment. The research team will conduct a data completion check after every session prior to releasing participants. They will also check for 3-days post survey completion and follow-up with participants to complete the surveys if needed.

Quality control procedures will be implemented, beginning with the data entry system design and weekly validation of the data. Data entry on the EDC will have field-type validation (i.e. only numbers can be entered for number fields), range checks (to prevent out-of-range data entry), required fields and signatures (to prevent data incompleteness) and a lock feature after completion to prevent editing after data entry. Research staff will check for data completion every week. Any missing data or data anomalies will be reported in a note-to-file document (containing an explanation for data anomaly/missing data, and future corrections for prevention) and the anomaly will be fixed on the source record.

### Statistical methods

#### Statistical analyses and power calculations

Since this is the first trial examining the effects of microdosing psilocybin to alleviate symptoms of MDD, we will not use other comparable effect sizes from the microdosing literature. It also does not seem appropriate to assume that microdosing would have the same effect size as studies that use ten times this study’s dose. Instead, we expect the size and directionality of the effects observed from a brief microdosing regimen to reflect those of a mindfulness intervention. We subsequently calculated our expected power based on a small-to-medium effect size and assuming a small random variance in the outcome measures. We used the patient-health questionnaire (PHQ-9; see Supplementary material C) as our main predictor as its psychometrics are well-studied. In addition to the small random variance in the scores, we also predicted a variance of 0.16 in PHQ-9 scores.^51^ We ran a power analysis using a hierarchical linear model in R, version 3.6.0 for Windows, with the PHQ-9 as our main predictor (see R code in Supplementary material C). This power analysis uses a frequentist method to determine whether this sample will be able to detect a significant difference in the slopes between the placebo-first and psilocybin-first groups just before crossover to open label and at the end of the experimental phase of the study. It uses a linear effects model to assess the Group × Session interaction on weekly PHQ-9 scores. This model used the lme4 package using restricted maximum likelihood. It included fixed effects (Group, Session, Interaction) and random effects (participant-level intercepts and session slopes). The lmerTest package was used to compute the p-values for fixed effects using Satterthwaite’s approximation.

Power analysis output based on whether the weekly change in score is 1 point (Table 3) or 0.75 points (Table 4), in addition to small random variance and 0.16 point variance weekly. Group × Session means that this is the power to detect an interaction between group assignment (placebo or psilocybin first) and time if there is an effect after the final experimental session; Group × Session 5 means that this is the power to detect the same interaction after the fourth experimental session (before crossover).

**Table 3:** Power analysis for weekly change in PHQ = 1

Sample size (hypothesised *N*)	Group × Session (1 − *β*)	Group × Session 5 (1 − *β*)
20	0.86	0.96
30	1	1
40	1	1

PHQ, Patient Health Questionnaire.

**Table 4:** Power analysis for weekly change in PHQ = 0.75

Sample size	Group × Session (1 − *β*)	Group × Session 5 (1 − *β*)
20	0.62	0.77
30	0.86	0.98
40	0.93	0.99

PHQ, Patient Health Questionnaire.

The two outputs in Tables 3 and 4 show different possible weekly changes in PHQ-9 scores (1 or 0.75). Even with a relatively small (0.75) change, we would still have 99% power to detect an effect just before crossover (Group × Session 5).

### Sub-group data analysis and handling missing data

Participants who completed at least one dosing session and one follow-up survey session will be included in the final analysis. Participants who decline to respond to a measure will be withdrawn from the trial for non-compliance. If participant data is missing due to technical issues, we will use multiple imputation to interpolate participant scores based on previous responses to the same measure.^52^

### Data and safety monitoring

Due to budget constraints, a Data Monitoring Committee could not be hired as such spending would affect other areas like participant recruitment and treatment procedures. Alternatively, an independent clinical monitor will be hired and will schedule visits at the beginning, midpoint and end of the study to assess rate of enrolment and study compliance with GCP guidelines. The monitor will also ensure appropriate documentation and complete and accurate reporting of CRFs (including consent documentation), study data and investigational product accountability logs. An interim Monitoring Visit Report will be initiated and shared with the study team to determine protocol adherence, patient safety, and data completion and minor corrective actions if any. The monitor will report to the principal investigator, who will be tasked with overseeing trial progress, adherence, participant safety and development of new information, in addition to maintaining the quality of study conduct through ongoing data monitoring.

The investigational site will be audited and inspected at random; the investigator will provide direct access to all trial-related sites, source data/documents and reports for monitoring and auditing and inspection by local and regulatory authorities.

### Adverse event reporting and harms

During each study visit, the principal investigator will assess and record (if any) adverse events with the participant. Adverse events will be described by symptoms/signs, severity, duration, outcome and relation to study drug, in line with The National Cancer Institute’s Common Terminology Criteria for Adverse Events (CTCAE) Version 3.0.

If participants report worsening symptoms or suicidality during the study or follow-up, the study psychiatrist will assess the participant and refer them for urgent psychiatric care and/or to an emergency department if necessary. If a participant develops a severe MDD episode, they will be withdrawn from the study and referred to appropriate medical care.. All serious adverse events (SAEs) will be followed until satisfactory resolution or until the principal investigator deems the event to be chronic or the participant is stable. The principal investigator will report adverse events and SAEs to Health Canada in accordance with ICH/Tri-Council Policy Statement (TCPS-2) guidelines.

### Ethics

The present clinical trial will be conducted in accordance with ICH, GCP, Health Canada Division 5, the Declaration of Helsinki, the Health Canada and Public Health Agency of Canada (PHAC) Research Ethics Board (REB) and applicable SOPs. All personnel involved in this study have completed Human Subjects Protection, ICH and GCP training.

### Data access

The final anonymised data-set will be made open to the public, in compliance with the *Tri-Agency Open Access Policy on Publications*, via Open Science Framework (https://osf.io/). There are no contractual agreements that limit access for investigators or the general public. The data and pre-registrations will be shared to whatever extent possible using a Creative Commons BY-NC-SA 4.0 licence.

#### Dissemination policy

The results of the primary outcome of this trial will be published as a manuscript in a peer-reviewed science or medical journal regardless of the magnitude or direction of effect. The study’s secondary outcomes may be published within the same or separate manuscripts in peer-reviewed journals. The results of all manuscripts will be in accordance with the planned pre-registered analyses. The criteria for authorship for all manuscripts will be in accordance with the International Committee of Medical Journal Editors recommendations. The results of this trial may also be shared at science conferences and through the media.

#### Ancillary and post trial care

In case a participant experiences an effect related to IP consumption during a study visit, the research team will inform the psychiatrist and support the participant in managing symptoms. The research team will only assist the participant within their qualifications. If a participant experiences psychological distress, panic or anxiety, the research team will remind them that they have taken a psychoactive drug and will receive psychological support from a trained staff member. The research team will remain with the participant 5 h after IP administration or until the participant is deemed stable by physician evaluation and sobriety tests. The principal investigator will follow up with participants to manage symptoms if they persist beyond the study visit. If applicable, participants will be referred to an appropriate healthcare provider.

At study completion or day of withdrawal, participants will be given an opportunity to request a referral for further therapeutic or medical care. Participants will also be provided with an Exit Plan that includes a summary of treatments completed, current medications and the study team’s contact information. Participants who fail screening will be sent an ineligibility email containing resources for mental health services.

## Discussion

This paper describes the detailed procedures that will be undertaken to study the effects of microdosing psilocybin for MDD. To our knowledge, this will be the first and largest microdosing study on psilocybin for depression. In contrast to traditional therapeutic models, which have only a 50% response rate and unwanted side effects, psychedelic use shows promise for treating mood disorders. However, psychedelics’ induction of hallucinogenic states may be undesirable for individuals with MDD. Microdosing is an appealing model that has been shown to improve mood and health while circumventing hallucinogenic effects, though studies on microdosing have been observational in nature. We aim to address this gap with our double-blind, placebo-controlled microdosing regimen to primarily test the safety and tolerability of microdosing psilocybin, and its effects on mood and well-being, over a period of 9 weeks to up to 2 years.

## Supporting information

Beidas et al. supplementary material Beidas et al. supplementary material

## Data Availability

Data availability is not applicable to this article as no new data were created or analysed in this study.

## Supplementary material

The supplementary material is available online at https://doi.org/10.1192/bjo.2025.10968
