# Microdosing psilocybin for major depressive disorder: study protocol for a phase II double-blind placebo-controlled randomised partial crossover trial – CORRIGENDUM

**DOI:** 10.1192/bjo.2026.12000

**Published:** 2026-05-13

**Authors:** Zeina Beidas, Anya Ragnhildstveit, Adam Blackman, Thomas Anderson, Emily Fewster, Omer A. Syed, Valentyne Sobolenko, Ismail Kaan Kanca, Tatiana Son, Norman Farb, Rotem Petranker

In the original publication of this article, some of the supplementary material was missing by mistake.

This has since been added to the article, and this correction published.

## Supporting information

10.1192/bjo.2026.12000.sm001 Beidas et al. supplementary material Beidas et al. supplementary material
