# Supplementary material for: Microdosing psilocybin for major depressive disorder: study protocol for a phase II double-blind placebo-controlled randomised partial crossover trial
Source: BJPsych Open. 2026 Feb 16;12(2):e65. doi: 10.1192/bjo.2025.10968 (PMC12926881; doi:10.1192/bjo.2025.10968)
Supplement: Beidas et al. supplementary material [file S205647242510968Xsup001.docx]

| **Appendix A: A full list of outcome measures.** | | | |
| --- | --- | --- | --- |
| **Screening Measures** | | | |
| **Instrument** | **Domain** | **Type** | **Description** |
| Columbia Suicide severity Rating Scale (C-SSRS) [1] | Mood: suicidal ideation, ideation intensity, lethality, and behaviour. | Clinician-reported | Assessment measuring four constructs across four subscales, including 1) severity of ideation, rated on a 5-point ordinal scale in which 1=wish to be dead, 2=nonspecific active suicidal thoughts, 3=suicidal thoughts with methods, 4=suicidal intent, and 5=suicidal intent with plan, 2) the intensity of ideation, which comprises 5 items, each rated on a 5-point ordinal scale: frequency, duration, controllability, deterrents, and reason for ideation, 3) behaviour, rated on a nominal scale that includes actual, aborted, and interrupted attempts; preparatory behaviour; and non-suicidal self-injurious behavior and 4) lethality, rated on a 6-point ordinal scale, and if actual lethality is zero, potential lethality of attempts is rated on a 3-point ordinal scale. A “yes” response to any one of the ten suicidal ideation and behaviour questions is considered as endpoint for positive suicidal ideation or behaviour. |
| Vital Signs | Safety: blood pressure, heart rate, and pulse | Clinician-report | Assessment measuring blood pressures, heart rate, and pulse. Each assessment visit will involve one or two readings. To qualify for the study, participants must have at least one reading not exceeding 150 systolic and 90 diastolic. Significant increases or decreases in blood pressure following baseline will be considered an AE. |
| **Primary Measures** | | | |
| **Instrument** | **Domain** | **Type** | **Description** |
| Patient Health Questionnaire Somatic-Anxiety-Depression (PHQ-SADS) [2] | Mood: Somatic, Anxiety and Depression Symptoms | Self-reported | A 32-item measure including 9-items assessing depressive symptoms, 7 items assessing anxiety and 15 items for somatic symptoms. Depressive symptoms are evaluated using a 4 point Likert scale, with scores used to classify depression severity as follows: None (0-4), Minimal (5-9), Moderate (10-14), Moderately severe (15-19), and Severe (20-27)). Frequency of anxiety is rated on a 4-point Likert scale (0=Not at all to 3=Nearly every day) with scores of 5, 10, and 15 represent cut-off points for mild, moderate, and severe anxiety, respectively. Somatic symptoms are evaluated using a 3 point Linkert scale (0=Not bothered to 2=Bothered a lot) with scores of 5, 10, and 15 used as cutoff points to classify somatic symptoms as either low, medium, or high, respectively. |
| Structured Clinical Interview for DSM-5 (SCID-5) [3] | Mood: Mood Disorders Symptoms | Clinician-reported | Only the SCID Screening Module and the Current Major Depressive Episode section of the instrument were used.  If participant affirmed the presence of any disorders during this screening, clinician proceeded to the relevant section. However, given the exclusion criteria for the study included major co-morbid disorders, this was used as an additional measure, and “No” was an expected answer to all of the screening questions. The evaluation of presence of a major depressive episode involved a degree of clinical judgment made by an experienced professional, with comprehensive notes on participants' responses recorded. |
| **Secondary Measures** | | | |
| **Instrument** | **Domain** | **Type** | **Description** |
| Quick Inventory Of Depressive Symptomatology [4] | Mood: Depression | Self-reported | Clinical assessment tool designed to measure the severity and changes in depressive symptoms in individuals, consisting of 16 items that assess nine domains of depression, including mood, sleep disturbance, energy level, concentration, psychomotor ability, guilt, weight, interest, and suicidal ideation. The highest score of all items measuring each of the sleep, weight, and psychomotor domain will be obtained and added with the rest of the 6 scores on the depressive symptoms domains. Total scores range from 0-27, with higher scores indicating higher severity in depressive symptoms. Scores 6 to 10 indicate mild depression, 11 to 15 moderate depression, 16 to 20 severe and scores greater than 21 indicate very severe depression. |
| Generalized Anxiety Disorder 7 (GAD-7) [5] | Mood: Anxiety | Self-Reported | 7-item scale comprising a single factor; items rated on a 4-point scale, with ‘not at all’ (0) and ‘nearly every day’ (3) as endpoints; scored by summing all items together; total scores range from 0 to 21; higher scores reflect greater anxiety. |
| Sustained Attention Reaction Task (SART) [6] | Attention: Sustained Attention | Self-Reported | Go/No-go paradigm with random series of single digits are presented. Participants are instructed to press a response key following each presentation with the exception of a designated “no-go” digit. Scoring is assessed by recording reaction time and error rate during 4 blocks (20 minutes total). |
| Unusual Uses Task (UUT) [7] | Creativity: Divergent Thinking | Self-Reported | The task requires that participants generate creative uses for mundane objects. Participants are asked to write down the three most unusual, creative, and uncommon uses for a single item (e.g. brick, knife), allotted 5 minutes in total. Responses are rated across three dimensions: uncommon, clever, and remote. |
| Remote Associates Task (RAT) [8] | Creativity: Convergent Thinking | Self-Reported | Participants are shown a set of three related words (e.g. show, life, row) and asked to identify a fourth associated word – which can be related either by forming a compound word, a common phrase or by close semantic association (e.g. boat). Each answer is scored as either correct or incorrect, and the total score is taken as the number of correct answers. The task consists of 30 word sets, presented one at a time for 15 seconds |
| Five Dot Problem (5-Dot) [9] | Creativity: Non-Verbal Fluency and Divergent Thinking | Self-Reported | Participants are presented with a grid of rectangles; for each grid (9 dots), participants are instructed to connect any two or more dots using smooth, connected lines. Participants are asked to produce as many different figures as possible in the 5-minute time limit. Total score consists of a count of: the number of figures, the number of repetitions, the number of rotated figures, the number of figures with added dots, the number of self-corrections, and the percentage of figures that are correct |
| Insight Problems [10] | Creativity: Problem Solving | Self-Reported | The measure consists of 6 sets of short word or diagrammatic problems that participants must solve. Each problem has a unique solution that is scored as correct or incorrect. Participants are given 5 minutes to answer all problems. |
| Reading the Mind in the Eyes Test (Mind In Eyes) [11] | Pro-sociality: Social Cognition | Self-reported | The participant is shown 36 grey-scale photographs that depict only the eye region of the face. They are required to rate, by choosing among four words that describe mental states, what the person in the photo is thinking or feeling. Only one of the answers is deemed correct, and the test is scored by counting correct and incorrect responses. |
| Metronome Response Task (MRT) [12] | Attention: Sustained attention | Self-reported | During the task, participants are presented with a series of auditory tones (one every 1300 ms) and instructed to respond with a button press synchronously to each presentation. The primary measure is the latency between the presentation of the tone and the participant’s response. The task also assesses participant reports of mind wandering with intermittent “thought probes” which require the participant to report on the content of their thoughts. |
| Finger-to-Nose Test (FTN) [13] | Sobriety | Clinician-reported | The participant is instructed to stand facing the examiner extending their index finger to touch the examiner’s index finger on the right. They are then asked to touch their nose then touch the examiner’s index finger in the, repeating the cycle on the left. The full index finger-to nose- to index finger round is repeated from left to right, then twice again with the eyes closed when touching the nose. . Every move (finger to nose on right, middle, left, and back) with eyes open and closed, is measured as either pass or fail based on signs of tremor or difficulty controlling the range of motion. All parts must be scored as “pass” for participant to be considered sober. |
| Balance Test (Romberg Test) [14] | Sobriety | Clinician-reported | Participants are instructed to bring their feet together and hands by their side with eyes open for 30 seconds, followed by 30 seconds of eyes closed. Examiner evaluates participant’s ability to maintain balance while standing Impairment is judged by: failure to keep the eyes closed, a loss of balance requiring the feet to move, falling, or inability to stand upright with eyes with minimal swaying. The “eyes closed” and “eyes open” must be recorded “pass” for the participant to be considered sober. |
| Standardized Field Sobriety Tests (SFST) [15] | Sobriety | Clinician-report | The test is administered by a trained experimenter and includes three components: the Horizontal Gaze Nystagmus (HGN), the Walk-and-Turn (WAT), and the One-Leg Stand (OLS) assessing participants impairment based on balance, and eye movement. Typically, participants are classified as impaired overall whenever they show impairments on two out of three SFST. |
| Big Five Inventory II (BFI-II) [16] | Personality: Extraversion, Negative Emotionality, Conscientiousness, Agreeableness, and Open-Mindedness | Self-reported | Five-factor measure of personality consisting of 60 short, descriptive items that are rated on a 5 point Linkert scale (1=Disagree strongly to 5=Agree strongly). It is divided into 15 4-item facet scales that aggregate into 5 12-item domain scales. |
| **Subjective Measures** | | | |
| **Instrument** | **Domain** | **Type** | **Description** |
| Five Facet Mindfulness Questionnaire (FFMQ) [17] | Mindfulness: observing, describing, acting with awareness, non-judging, and nonreactivity | Self-reported | 39-item self-report questionnaire that measures five sub-scales of mindfulness: observing (8 items), describing (8 items), acting with awareness (8 items), non-judging (8 items), and nonreactivity (7 items). Respondents rate the degree to which each statement is true for them on a 5-point Linkert-type scale (1=never or very rare true 5=very often or always true; some items use a reverse-scoring). The scores of all subscales are added to give a total measure of mindfulness. |
| Multidimensional Assessment of Interoceptive Awareness - Version 2 (MAIA-2) [18] | Wellbeing: Interoceptive Awareness | Self-reported | 37-item self-report state-trait questionnaire designed to measure multiple dimensions of interoception. Statements are rated using a 5 point Likert-type scale (0=Never to 5=Always; some items use a reverse scoring) comprised of 8 factor subscales: Noticing (4 items), Not-Distracting (6 items), Not-Worrying (5 items), Attention Regulation (7 items), Emotional Awareness (5 items), Self-Regulation (4 items), Body Listening (3 items), and Trust (3 items). The outcome of each subscale is obtained by taking the average score of the items in each subscale. |
| Mystical Experience Questionnaire, 30-item (MEQ30) [19] | Experience: Mystical, Positive Mood, Transcendence of Time and Space, and Ineffability | Self-reported | A 30-item scale with four factors of mystical experiences: mystical, positive mood, transcendence of time and space, and ineffability. The mystical factor includes items from the internal unity, external unity, noetic quality, and sacredness scales. The items are rated on a 6-point Likert-type scale, where 0=“none; not at all,” 1=“so slight cannot decide,” 2=“slight,” 3=“moderate,” 4=“strong and 5=“extreme. Scale scores are the sum of all responses on a given scale and a “complete mystical experience” is defined as a score ≥60% of the total possible score on each subscale. |
| Inclusion of Others in the Self Scale (IOS) [20] | Pro-sociality: Feelings of closeness | Self-reported | A single item measure with six Venn-like diagrams of varying degrees of overlap (1=no overlap, 2= little overlap, 3= some overlap, 4= equal overlap, 5=strong overlap, 6= very strong overlap, 7= most overlap, measuring the closeness participants currently feel with their: (1) Future self; (2) Past self; (3) Friends / Coworkers; (4) a stranger in the street; (5) Family (6) Romantic Partner. The number given on each diagram is the participant’s score. |
| Single Item Sleep Quality Scale (SQS) [21] | Well-being: Sleep Quality | Self-reported | A single item measure of quality of sleep over a 7 day period using a visual-analog scale, scored from 0-10 (0=terrible, 1-3=poor, 4-6=fair, 7-9=good and 10=excellent). |
| Positive and Negative Affective Scale (PANAS) [22] | Mood: negative affect, positive affect | Self-reported | Two 10-item mood scales containing words that describe different feelings and emotions (e.g., upset, enthusiastic). Participants indicate to what extent they felt each of the emotions on that day, with response options ranging between (very slightly or not at all) to (extremely). Scores are summed across each of the 10 items to yield separate scores for negative affect (NA) and positive affect (PA). |
| Qualitative Reports (QR-SB, QR-SPD, QR-SR) | Qualitative experiences: social perceptions, symptoms, and subjective experience | Self-reported | A set of open-ended questions evaluating responses covering the respondents’ experience in the trial over 3 domains: QR-SB (social perceptions and sharing, 3 questions), QR-SPD (self-assessed symptoms and physiological discomfort, 3 questions), and QR-SR (individual experience/subjective responses, 8 questions). |
| Brief Pain Inventory, Short Form (BPI-SF) [23] | Mood: Pain | Self-reported | Scale of pain intensity (4 items: current, worst, least, and average) and pain interference (7 items: general activity, mood, walking ability, normal work, social relationships, sleep, enjoyment of life). Each item measured on a 10-point scale, with average pain intensity is scored using cut-offs: no or mild pain (0-2), moderate pain (3-5), severe pain (6-10). Also includes questions about the location of pain, current treatments or medications and the percentage of pain relief obtained from them. |
| Quality of Life Inventory (QOLI) [24] | Wellbeing: Satisfaction | Self-reported | 32-item scale with 16 domains: health, self-esteem, goals and values, money, work, play, learning, creativity, helping, love, friends, children, relatives, home, neighbourhood, and community. Each domain evaluated based on its importance (three point Linkert scale from 0 to 2), and satisfaction (six point Linkert scale, from −3 to +3). Total score is computed by dividing the sum of the domain scores by the number of non-zero domain scores, ranging from -6 to +6. |
| GSE (General Self Efficacy Scale) [25] | Wellbeing: Self-efficacy | Self-report | A 10-item measure, where all statements are rated on a 4-point Likert-type scale (ranging from 1=Not at all to 4=Exactly true). The total score is the sum of all items (range 10-40). |
| Dysfunctional Attitudes Scale, 17 item (DAS-A-17) [26] | Wellbeing: Perfectionism and dependency | Self-report | A 17-item scale, items rated on a 7-point Likert scale. The scale includes a total score and two subscales: perfectionism/performance evaluation (11 items) and dependency (6 items). The total score is the sum of the 17-items (range: 17–119) with higher scores indicating more dysfunctional attitudes. |

**References**

1. Posner K, Brent D, Lucas C, Gould M, Stanley B, Brown G, Fisher P, Zelazny J, Burke A, Oquendo M, Mann J. Columbia-suicide severity rating scale (C-SSRS). Columbia University Medical Center 2008.
2. Kroenke K, Spitzer RL, Williams JB, Löwe B. The patient health questionnaire somatic, anxiety, and depressive symptom scales: a systematic review. General hospital psychiatry 2010; 32(4): 345-359.
3. First MB, Williams JBW, Karg RS, Spitzer RL. Structured clinical interview for DSM-5 disorders: SCID-5-CV clinician version. Arlington, VA: American Psychiatric Association Publishing; 2016.
4. Rush AJ, Trivedi MH, Ibrahim HM, Carmody TJ, Arnow B, Klein DN. The 16-Item quick inventory of depressive symptomatology (QIDS), clinician rating (QIDS-C), and self-report (QIDS-SR): A psychometric evaluation in patients with chronic major depression. Biological Psychiatry 2003; 54(5): 573–583.
5. Spitzer RL, Kroenke K, Williams JB, Löwe B. A brief measure for assessing generalized anxiety disorder: the GAD-7. Arch Intern Med 2006; 166(10):1092-7.
6. Robertson IH, Manly T, Andrade J, Baddeley BT, Yiend J. `Oops!’: Performance correlates of everyday attentional failures in traumatic brain injured and normal subjects. Neuropsychologia 1997; 35(6): 747–758.
7. Silvia PJ, Winterstein BP, Willse JT, Barona CM, Cram JT, Hess KI, Martinez JL, Richard CA. Assessing creativity with divergent thinking tasks: Exploring the reliability and validity of new subjective scoring methods. Psychology of Aesthetics Creativity and the Arts 2008; 2(2): 68–85.
8. Mednick MT, Halpern S. Remote associates test. Psychological Review 1968.
9. Regard M, Strauss E, Knapp P. Children’s Production on Verbal and Non-Verbal Fluency Tasks. Perceptual and Motor Skills 1982; 55(3): 839–844.
10. Beveridge M, Parkins E. Visual representation in analogical problem solving. Memory & Cognition. 1987 May; 15(3): 230–7. doi:10.3758/bf03197721
11. Baron-Cohen S, Wheelwright S, Hill J, Raste Y, Plumb I. The “Reading the Mind in the Eyes” Test revised version: a study with normal adults, and adults with Asperger syndrome or high-functioning autism. The Journal of Child Psychology and Psychiatry and Allied Disciplines 2001; 42(2): 241-251.
12. Seli P, Cheyne JA, Smilek D. Wandering minds and wavering rhythms: Linking mind wandering and behavioral variability. Journal of Experimental Psychology: Human Perception and Performance 2013; 39(1): 1–5.
13. Swaine BR, Desrosiers J, Bourbonnais D, Larochelle JL. Norms for 15 to 34-Year-Olds for Different Versions of the Finger-to-Nose Test. Archives of Physical Medicine and Rehabilitation 2005;  86(8): 1665–1669.
14. Bickley LS, Szilagyi PG, Bates B. In Bates’ guide to physical examination and history taking: 718. Lippincott Williams & Wilkins, 2009.
15. Tharp V, Burns M, Moskowitz H.  Development and field test of psychophysical tests for DWI arrest. NHTSA, 1981.
16. Soto CJ, John OP. The next Big Five Inventory (BFI-2): Developing and assessing a hierarchical model with 15 facets to enhance bandwidth, fidelity, and predictive power. Journal of personality and social psychology. J Pers Soc Psychol 2017; 113(1): 117-143.
17. Baer RA, Smith GT, Hopkins J, Krietemeyer J, Toney L. Five facet mindfulness questionnaire. Assessment, 13(1), 27-45.
18. Mehling WE, Acree M, Stewart A, Silas J, Jones A. The Multidimensional Assessment of Interoceptive Awareness, Version 2 (MAIA-2). PLOS ONE 2018; 13(12): e0208034.
19. MacLean KA, Leoutsakos JM, Johnson MW, Griffiths RR. (2012) Factor analysis of the Mystical Experience Questionnaire: a study of experiences occasioned by the hallucinogen psilocybin. J Scient Study Religion 2012; 51: 721–737.
20. Aron A, Aron EN, Smollan D. Inclusion of other in the self scale and the structure of interpersonal closeness. Journal of personality and social psychology 1992; 63(4): 596.
21. Snyder E, Cai B, DeMuro C, Morrison MF, Ball W. A new single-item sleep quality scale: results of psychometric evaluation in patients with chronic primary insomnia and depression. Journal of Clinical Sleep Medicine 2018; 14(11): 1849-1857.
22. Watson D, Clark LA, Tellegen A. Development and validation of brief measures of positive and negative affect: the PANAS scales. Journal of personality and social psychology 1988; 54(6): 1063-1070.
23. Tan G, Jensen MP, Thornby JI, Shanti BF. Validation of the brief pain inventory for chronic nonmalignant pain. The Journal of Pain 2004; 5(2): 133–137.
24. Frisch MB. QOLI: Quality of life inventory: Manual and treatment guide. Pearson San Antonio, 1994.
25. Schwarzer R, Jerusalem M, Weinman J, Wright S, Johnston M. Generalized Self-Efficacy Scale. Measures in Health Psychology. NFER-NELSON, 35–37, 1995.
26. Weissman AN, Beck AT. Development and validation of the Dysfunctional Attitude Scale: A preliminary investigation. ERIC 1978; ED167619.
